# Spatio-Temporal Clustering of Hand, Foot, and Mouth Disease at the County Level in Guangxi, China

**DOI:** 10.1371/journal.pone.0088065

**Published:** 2014-02-05

**Authors:** Yi-hong Xie, Virasakdi Chongsuvivatwong, Zhenzhu Tang, Edward B. McNeil, Yi Tan

**Affiliations:** 1 Guangxi Zhuang Autonomous Region Center for Disease Prevention and Control, Nanning, Guangxi, China; 2 Epidemiology Unit, Faculty of Medicine, Prince of Songkla University, HatYai, Songkla, Thailand; 3 International Field Epidemiology Training Program (IFETP), Bureau of Epidemiology, Ministry of Public Health, Bangkok, Thailand; University of Illinois at Chicago, United States of America

## Abstract

**Background:**

Amid numerous outbreaks of hand, foot and mouth disease (HFMD) in Asia over the past decade, studies on spatio-temporal clustering are limited. Without this information the distribution of severe cases assumed to be sporadic. We analyzed surveillance data with onset dates between 1 May 2008 to 31 October 2013 with the aim to document the spatio-temporal clustering of HFMD cases and severe cases at the county level.

**Methods:**

Purely temporal and purely spatial descriptive analyses were done. These were followed by a space-time scan statistic for the whole study period and by year to detect the high risk clusters based on a discrete Poisson model.

**Results:**

The annual incidence rate of HFMD in Guangxi increased whereas the severe cases peaked in 2010 and 2012. EV71 and CoxA16 were alternating viruses. Both HFMD cases and severe cases had a seasonal peak in April to July. The spatio-temporal cluster of HFMD cases were mainly detected in the northeastern, central and southwestern regions, among which three clusters were observed in Nanning, Liuzhou, Guilin city and their neighbouring areas lasting from 1.2 to 2.5 years. The clusters of severe cases were less consistent in location and included around 40–70% of all severe cases in each year.

**Conclusions:**

Both HFMD cases and severe cases occur in spatio-temporal clusters. The continuous epidemic in Nanning, Liuzhou, Guilin cities and their neighbouring areas and the clusters of severe cases indicate the need for further intensive surveillance.

## Introduction

Hand, foot and mouth disease (HFMD) is a common infectious disease mostly affecting children under 5 years of age. Over the past decade, many small and large outbreaks have been reported in Asia [1]–[8]. In China, HFMD has been a notifiable disease since May, 2008 after two consecutive years of large outbreaks affected more than 46,000 persons [9], [10]. Over six million cases had been reported up to the end of 2012 in China.

Previous studies in Singapore, Japan and Malaysia demonstrated that outbreaks of HFMD generally occurred in a 2–3 year cyclical pattern with strong seasonality [7], [8], [11] and the incidence was independently related to meteorological parameters such as temperature, humidity and rainfall [12]–[16]. The disease also has a global spatial variation [6]. Before 2000 only a few outbreaks occurred in different parts of the world [17]–[22], while numerous outbreaks have occurred in Asia over the last decade [1]–[6], [23]–[25], particularly in China since 2007. One study indicated that HFMD propagates in a composite space-time domain rather than having purely spatial or temporal variation [14]. Four spatio-temporal clusters had been detected at the provincial level in China [26].

Spatio-temporal clustering indicates the composite of place and time i.e. where and when the incidence is abnormally high. Identification of spatio-temporal clusters allows public health officials to understand the disease nature and launch timely surveillance and intervention programs at the correct site. However, most of the previous studies on spatial variation were separated from the temporal ones. Studies on smaller units (for example at the county level) and the spatio-temporal composite analyses are very limited [27], [28], and no studies on severe cases have been reported.

Guangxi is one of the serious HFMD epidemic provinces in China. A substantial increase in cases has been reported since 2008 with an incidence rate of 449.4 per 100,000 population reported in 2012. The number of severe cases (2,236) and fatalities (123) in that year was ranked first among 31 provinces in China. Identification of the spatio-temporal clustering of an outbreak, especially severe cases should lead to better allocation of medical resources and direct public health services planning. The objective of this study was to examine the spatio-temporal pattern of HFMD cases and severe cases at the county level in Guangxi, China.

## Methods

### Study area

**Figure 1:**
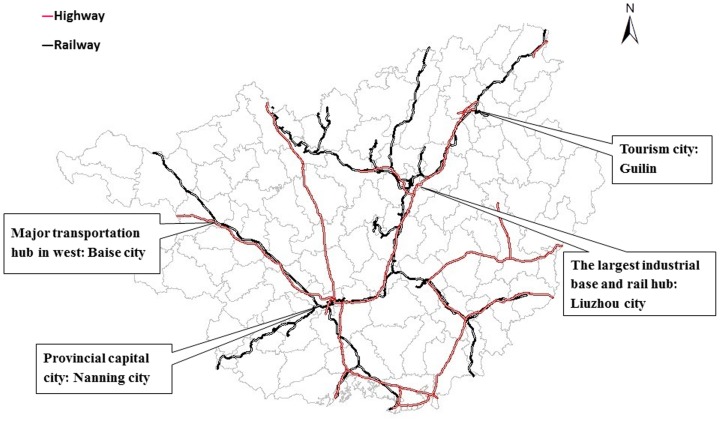
The distribution of main highways and railways in Guangxi and the location of four major transit centers.

Guangxi is located in southern China at latitude 20.54N to 26.24N and longitude 104.26E to 112.04E, which covers a total area of 236,700 km^2^ with a population of 46.5 million in 2012. There are 14 prefecture level divisions and 113 county level divisions in Guangxi. The population size at the county level ranges from 112,777 to 1,510,509 people. The distribution of main highways and railways in Guangxi are shown in Figure 1. There are four main cities (Baise, Nanning, Liuzhou and Guilin city), which are major transit centers linking the west to the northeast of the province.

### Data sources

The daily data of HFMD cases and severe cases form 1 May 2008 and 31 October 2013 were obtained from the China Information System for Disease Control and Prevention, which is an online, real-time surveillance system. All HFMD cases need to be reported to this system within 24 hours after diagnosis [29], [30] since HFMD was classified as a class “C” notifiable disease on May 2008 [31]. In Guangxi, this system covered all the hospitals at or above the township level. The information entered into the system includes name, age, gender, address, occupation, date of onset, date of diagnosis, type of diagnosis (clinical or lab confirmed), status (severe or non-severe), and laboratory test results. The diagnostic criteria of HFMD cases, severe cases and the procedure of laboratory test follow the HFMD Clinical Diagnosis and Treatment Guideline [32]–[34]. A severe case is defined as having fever, rash with blisters on the palms, soles and/or buttocks, and with neurological, respiratory or circulatory complications such as myoclonia, acute flaccid paralysis, encephalitis, cardiopulmonary failure or pulmonary edema. Each severe case must be confirmed by at least two experts at or above county level.

### Data analysis

Data management was done using R version 3.0.2. The incidence of all cases, severe cases and an analysis of pathogens over the 66 months were presented in graphs using R. The purely spatial distribution of HFMD cases and severe cases was shown in thematic maps using Quantum GIS.

A retrospective space-time scan statistic was applied to detect high risk clusters of HFMD cases and severe cases by using SaTScan™ software (version 9.1) with a discrete Poisson model. The space-time scan statistic is defined by a cylindrical window with a circular geographic base and with height corresponding to time [35]. The cylindrical window moves over space and time scanning for an elevated risk within the space-time window as compared to outside the window. The null hypothesis assumes that HFMD cases are randomly distributed. The alternative hypothesis for each scanning window is that there is an elevated risk inside the window as compared to outside [35]. The difference of the incidence inside and outside each window was calculated by the log likelihood ratio (LLR) [36]. Significant results (based on Monte Carlo simulation) from these were defined as a cluster. Among the statistically significant clusters, the cluster with the maximum LLR indicates one that is least likely to have occurred by chance which is thus the most likely cluster. Secondary clusters were those in rank order after the most likely cluster, based on their likelihood ratio test statistic. The relative risk of each cluster is the ratio of the estimated risk within the cluster to that outside the cluster [35].

In this study, we initially scanned the entire study period from 1 May 2008 to 31 October 2013. Further yearly scans were done to observe changes in clustering and to control the time trend in the whole study period [35]. As there is no consensus and optimal maximum-size of the spatial cluster size setting [37], [38], we firstly used the recommended value of 50% of the population in the spatial window to avoid the pre-selection bias [35]. To explore small to middle-sized clusters, we conducted the scan again at every 10% interval down to 10%. The scan results of HFMD cases in the entire 66 month study period were demonstrated by the 50%, 20% and 10% values. The severe cases and yearly scan results were displayed using a value of 20% as it is believed to have more power to detect the true cluster [26], [38].

For each run, we set the number of Monte Carlo replications to 999. The height of the cylindrical window which reflects time was set to be 50% of the scan timeframe and the clusters reported without geographical overlap. R software was used to illustrate the spatio-temporal cylinders of the 66 months data. Quantum GIS was used to visualize the yearly scan results.

## Results

### Demographic characteristics

From 1 May 2008 to 31 October 2013, there were 796,072 HFMD cases notified in Guangxi with an annual incidence rate of 298.3/100,000 population. Among these cases, there were 5,636 (0.71%) severe cases reported which included 350 fatalities. The incidence rate in males (323/100,000 population) was higher than in females (217/100,000 population, P value<0.01). 68.4% of HFMD cases and 83.7% of fatalities were aged under 3 years. 19.8% of HFMD cases and 8.9% of fatalities were preschool attending children.

A total of 3,485 severe cases (314 fatalities) were confirmed by laboratory testing. Among the confirmed cases, EV71, CoxA16 and other enteroviruses accounted for 80.2%, 2.8% and 17.0% of severe cases and 93.0%, 1.6% and 5.4% of fatalities, respectively.

Table 1 summarizes the incidence and pathogens by year. The incidence rate of HFMD cases increased over time (P value for linear trend = 0.017) while severe cases peaked in 2010 and 2012. For pathogens, other enteroviruses appeared consistently in the background in all years. Among the distinct viruses, the predominant pathogen alternated between EV71 and CoxA16.

**Table 1:** Incidence and pathogen of HFMD in Guangxi, 1 May 2008 to 31 October 2013.

Year	Incidence		Severe cases	Deaths	Number of laboratory confirmed cases (%)			
	Number of cases	Rate (/10^5^)			Total^a^	EV71^b^	Cox A16^b^	Other^b^
2008†	27676	57.9	48	15	219 (28/191)	85 (38.8)	11 (5.0)	123 (56.2)
2009	45649	94.8	25	9	386 (14/372)	60 (15.5)	139 (36.0)	187 (48.5)
2010	164192	338.1	2591	160	2403 (1412/991)	1713 (71.3)	128 (5.3)	562 (23.4)
2011	141966	308.4	365	33	3027 (256/2771)	727 (24.0)	1380 (45.6)	920 (30.4)
2012	231984	449.4	2236	123	5421 (1622/3799)	3284 (60.6)	469 (8.7)	1668(30.8)
2013‡	184605	357.6	371	10	2358 (153/2205)	223 (9.5)	686 (29.1)	1449 (61.5)
**Total**	**796072**	**298.3**	**5636**	**350**	**13814 (3485/10329)**	**6092 (44.1)**	**2813 (20.4)**	**4909 (35.5)**

† From 1 May. ‡Until 31 October

a Numbers in brackets are the numbers of severe/non-severe cases among all the laboratory confirmed cases.

b Numbers in brackets are the percentage of positive cases among all the laboratory confirmed cases.

### Temporal dimension

**Figure 2:**
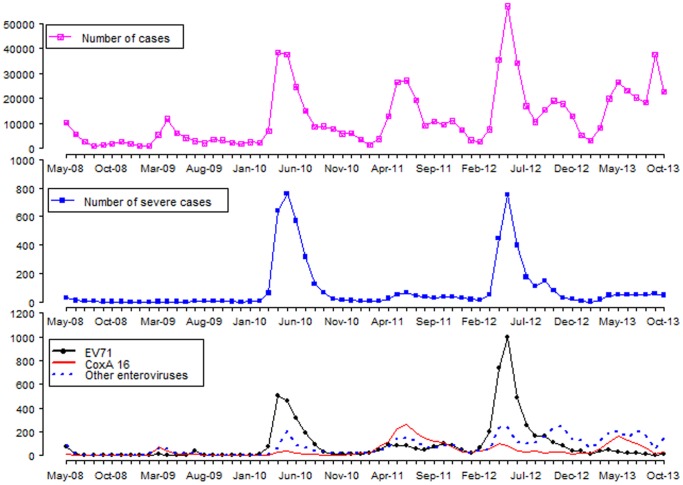
Monthly distributions of reported cases, severe cases and laboratory confirmed cases in Guangxi, China, from 1 May, 2008 to 31 Oct, 2013.

The monthly distribution of HFMD cases, severe cases and laboratory confirmed cases are illustrated in Figure 2. A general rising trend of HFMD cases was evident with clear seasonality and large and small alternating peaks. From 2008 to 2012, the first large peak occurred between April to July followed by smaller peaks in September to November. A different seasonal pattern was seen in 2013 when the epidemic started relatively late and lasted longer, with a higher peak occurring from August to October. The two distinct peaks of severe cases were seen from April to July. All viruses also had a seasonal pattern but with inconsistent predominant type. The peak of severe cases was consistent with the peak of EV71 confirmed cases. CoxA16 predominated only in 2011 whereas other enteroviruses appeared to co-oscillate with EV71 and CoxA16 in the beginning of the surveillance period and became relatively persistent after 2011. It is however, not possible to tell from these graphs whether the outbreaks were confined to certain areas only.

### Spatial dimension

**Figure 3:**
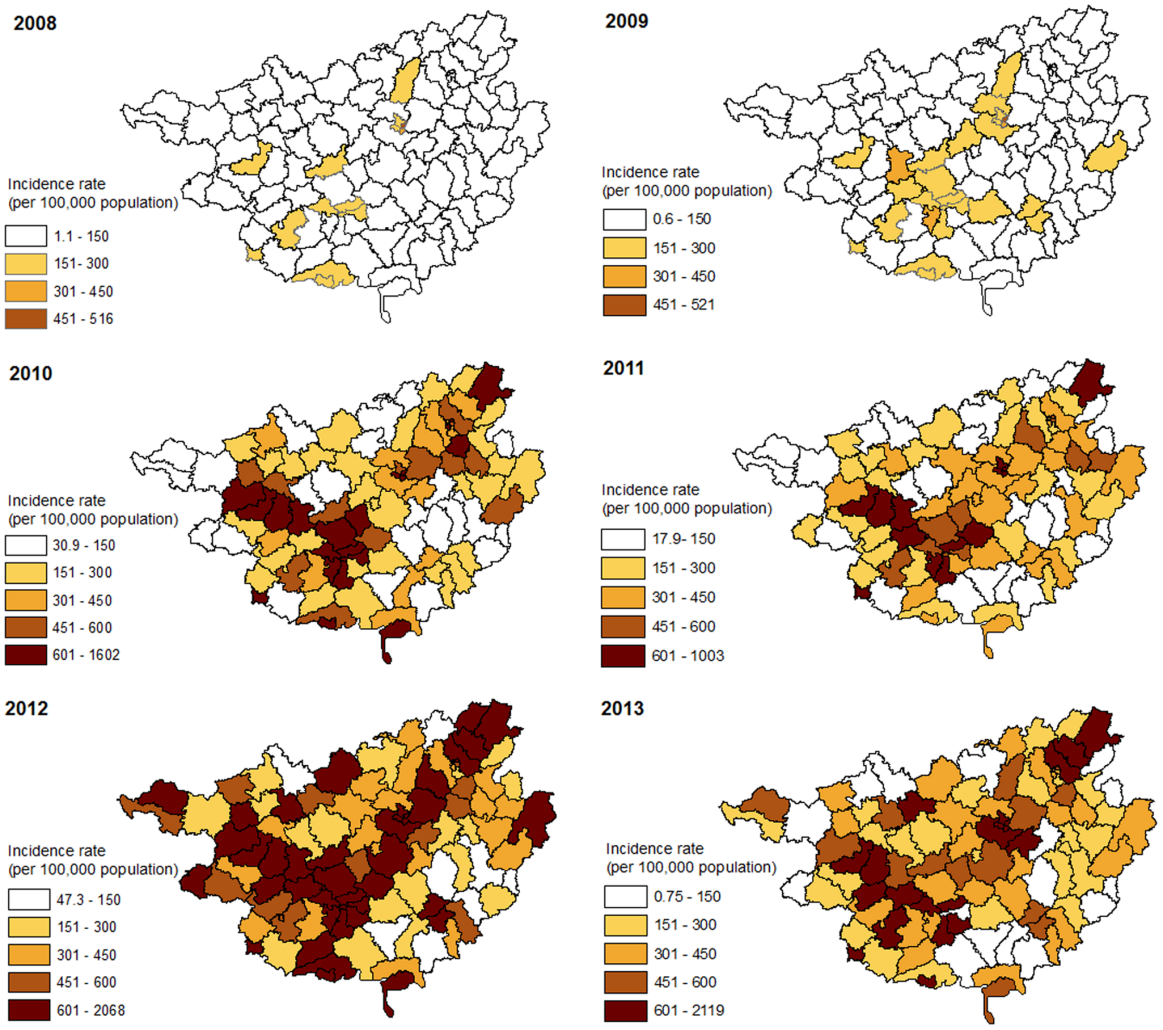
Yearly incidence rates (per 100,000 population) of HFMD at the county level in Guangxi, China, from 1 May, 2008 to 31 October, 2013.

The spatial distribution of HFMD cases by year is shown in Figure 3. Over the years, especially after the disease was well established in 2010, there were clear differences in the incidences among counties. Relatively high incidence rates appeared in the northeastern-southwestern belt whereas the northwestern and southeastern parts had relatively low incident rates. To check whether there was any temporal continuation of the increased incidence in certain areas, a spatio-temporal integration was done.

### Spatio-temporal analysis

**Figure 4:**
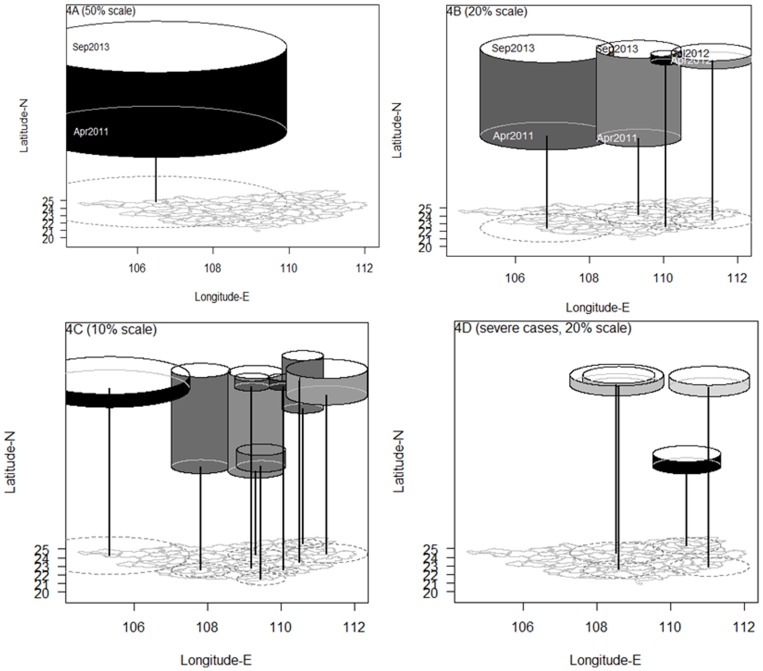
Spatio-temporal clusters of HFMD cases and severe cases in Guangxi, China from 1 May 2008 to 31 October 2013 (in the whole 66 month study period).

In the entire 66 month study period, one large cluster consisting of 56 counties centered in the west and lasting from April 2011 to September 2013 was detected when setting 50% of the population as the maximum spatial cluster size (Figure 4A). The cluster contained a considerable proportion of low risk areas when complemented by in Figure 3. When the maximum spatial cluster size was set to 20% of the population, four clusters were detected (Figure 4B), two of them lasting for approximately 2.5 years from April 2011 to September 2013 were located in the southwestern and central part of the province. Two shorter clusters were detected in the east and lasted 4 months from April to July 2012. When the maximum spatial cluster size was set to 10%, nine small clusters were detected (Figure 4C). Details of each of these clusters are shown in Table 2. The nine clusters varied in geographic and temporal sizes. The first cluster took place in the southern part of the province in the middle of 2010. Two long-lasting clusters located along the southwestern to northeastern belt, which included 10 counties around Nanning city and 11 counties around Liuzhou city started in 2011 and continued until 2013. Six clusters, five starting in April 2012 and one in May 2012, each lasted for a few months, except one which continued until the middle of 2013 (consisting of 8 counties around Guilin city). The most western occurring cluster consisted of 16 counties and had high intensity (RR = 5.39) but lasted only 3 months. In all of the 66 months, there were no clusters detected in 2008 and 2009 when the incidence was relatively low.

**Table 2:** Spatial-temporal clusters of HFMD in Guangxi, China, from 1 May 2008 to 31 October 2013 (setting 10% of the population as the maximum spatial cluster size).

Cluster period	Cluster center/Radius	Number of counties in the cluster	Cases per 10^5^ person years	RR^a^
1 May 2010-30 Sep 2010	(21.52 N, 109.46 E)/66 km	3	885	2.95
1 Mar 2011-30 Sep 2013*	(22.55 N, 107.81 E) /80 km	10	862	3.10
1 Apr 2011-30 Sep 2013	(24.33 N, 109.32 E)/76 km	11	695	2.45
1 Apr 2012-31 Jul 2012	(24.25 N, 105.33 E)/217 km	16	1589	5.39
1 Apr 2012-31 Jul 2013	(25.60 N, 110.59 E)/56 km	8	917	3.12
1 Apr 2012-30 Jun 2012	(22.54 N, 110.06 E)/41 km	4	1292	4.33
1 Apr 2012-30 Jul 2012	(22.82 N, 109.21 E)/48 km	3	1039	3.47
1 Apr 2012-30 Nov 2012	(24.49 N, 111.24 E)/111 km	10	637	2.13
1 May 2012-31 May 2012	(23.45 N, 110.51 E)/0 km	1	951	3.15

* The most likely cluster.

a All P-values<0.001. RR: relative risk.

For the severe cases, we illustrated only the 20% scale (Figure 4D). The first cluster occurred in 2010 in the northeast and included 17 counties around Guilin city with very high intensity (RR = 39.7) in 2010. The other three clusters in 2012 were detected in the southern (14 counties around Nanning), northern and central (21 counties around Liuzhou) and eastern (9 counties around Wuzhou) part of the province. All clusters occurred between April and July.

**Figure 5:**
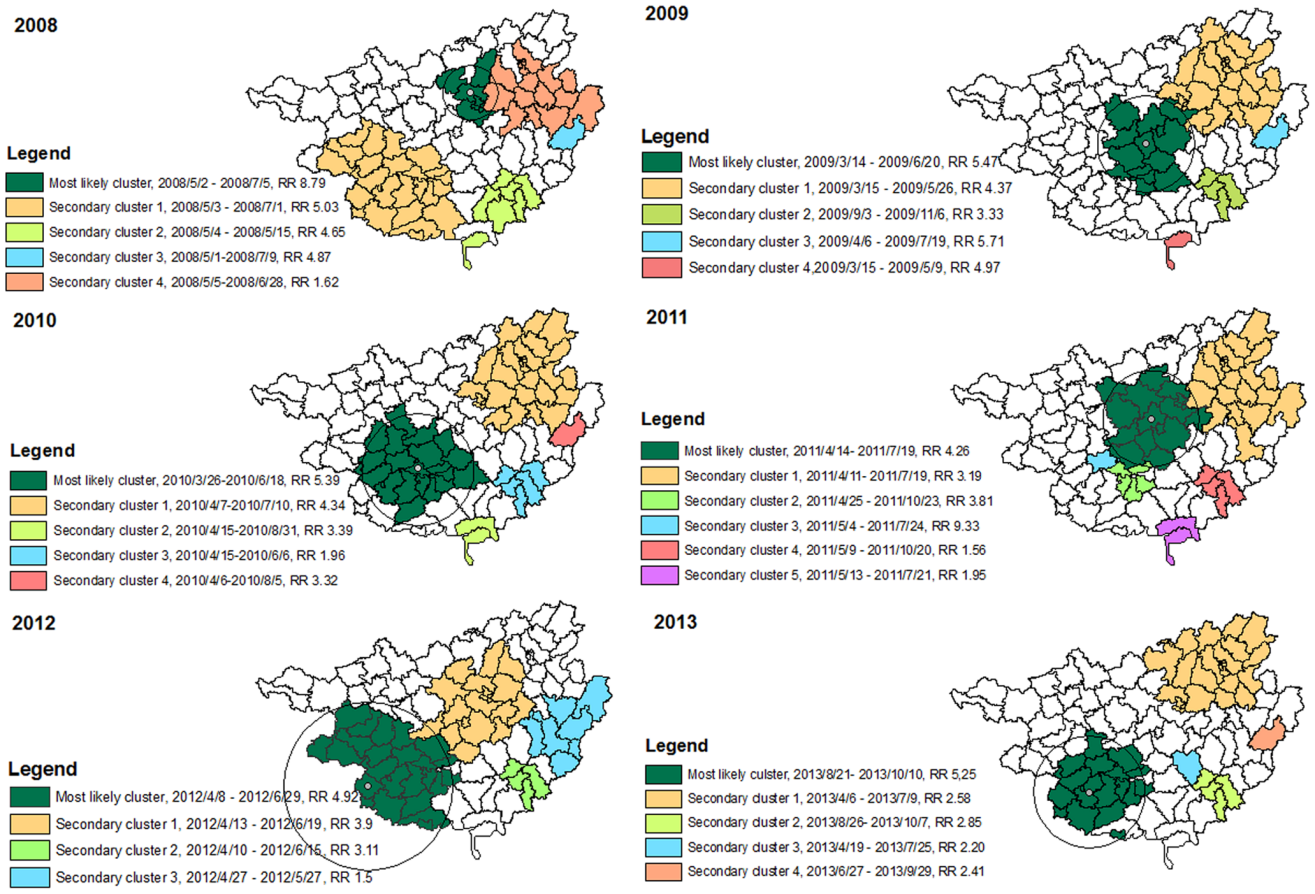
Spatio-temporal clusters of HFMD in Guangxi, China, from 1 May 2008 to 31 October 2013 (by year).

As the incidence of HFMD cases increased over time and the number of severe cases fluctuated each year, an analysis using the whole study period can only detect clusters in high incidence periods. To observe the cluster change and control for the temporal trend in the whole study period, scans were conducted in each year setting 20% of the population as the maximum spatial cluster size. The results are shown in Figure 5. In each year, apart from the most likely cluster, several secondary clusters with relative risk ranging from 1.5 to 9.3 were also detected. Most of clusters were located in the northeastern, central and southwestern parts of the province and occurred from April to July. There were also 2–3 small secondary clusters observed in the southeast. The most likely cluster in 2013 and four small secondary clusters in 2009, 2011 and 2013 occurred in the second peak period from August to October.

**Figure 6:**
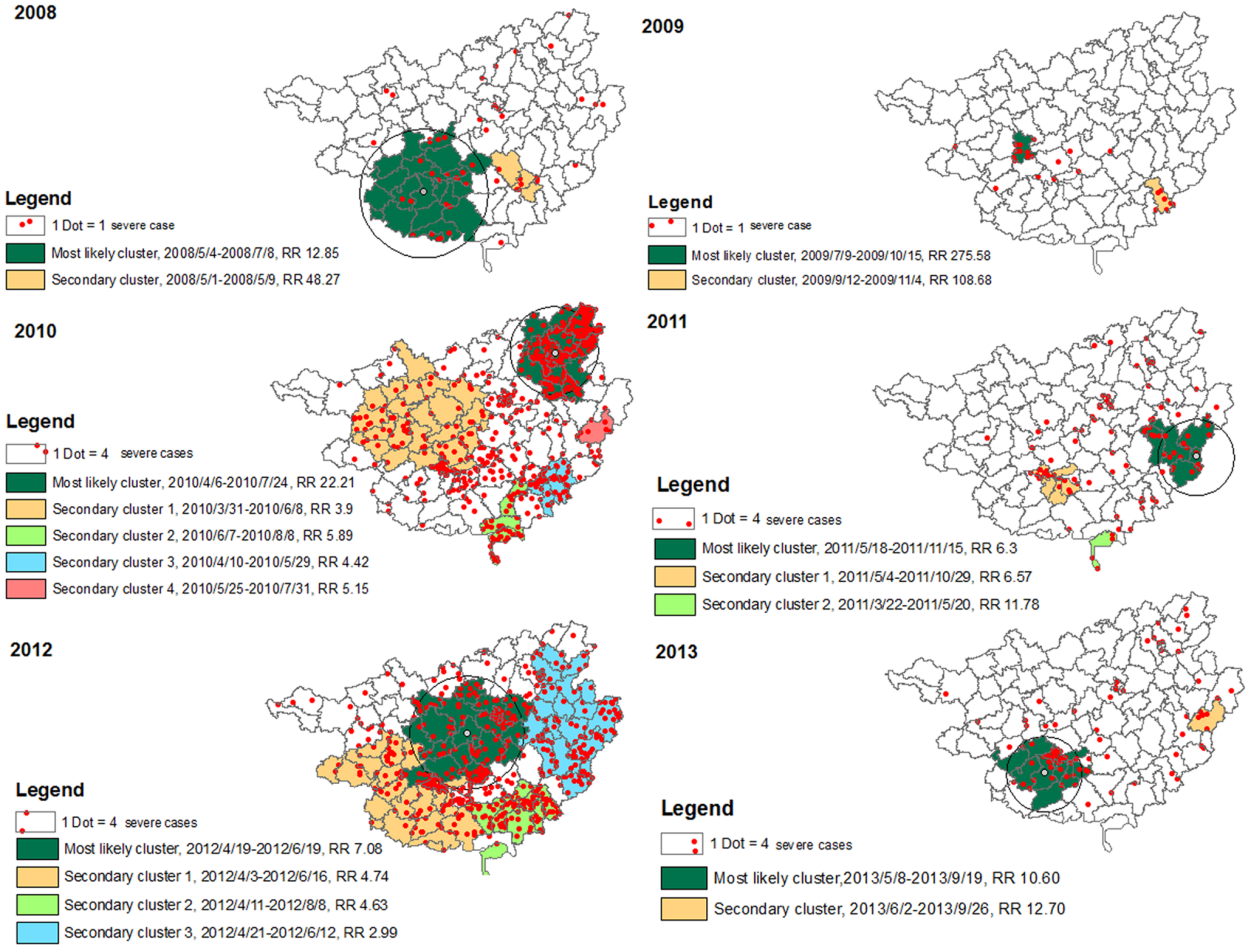
Spatial distribution (red dots) and spatio-temporal clusters of severe HFMD cases in Guangxi, China, from 1 May 2008 to 31 October 2013 (by year).

The yearly spatial distribution (red dots) and spatio-temporal cluster of severe cases are illustrated in Figure 6. There were 18 clusters detected. The most likely and secondary clusters of severe cases covered more than half of the counties in 2010 and 2012 when thousands of severe cases occurred. The cluster sizes were relatively small and the locations were less consistent in the other years. The severe cases included in the clusters accounted for 52.0%, 68.0%, 63.6%, 40.5%, 54.3% and 40.0% of all cases in the 6 years from 2008 to 2013, respectively. Four cluster centers were repeated in two different years.

## Discussion

Under the increasing trend of HFMD in Guangxi, we have demonstrated that both HFMD cases and severe cases had a spatio-temporal cluster pattern. For HFMD cases, in addition to seasonality (April to July), spatio-temporal analyse revealed that certain clusters lasted longer than one year. These clusters were located in Nanning, Liuzhou, Guilin city and their neighbouring areas along the southwestern-northeastern belt. In each year, around 40%–70% of the severe cases were aggregated within the clusters.

The incidence rate of HFMD in Guangxi increased by more than a factor of five from 2008 to 2013, which was higher than the average level in China [39] and neighboring provinces of Guangdong [27] and Guizhou [40]. A similar situation occurred in Singapore between 2001 and 2007 [7]. Similar to previous studies from USA and Singapore [7], [41], the current study found that EV71 and CoxA16 had an alternating pattern. The predominant pathogen in 2010 and 2012 was EV71 while in 2011 it was CoxA16. Consistent with previous studies, EV71 was strongly associated with severity and fatality [9], [17], [42].

HFMD is strongly seasonal [6], [7], [12]. Around 70% of cases are reported during warmer seasons between June to October in USA [41], while a nation-wide outbreak occurred in the autumn of 2008 in Finland [43]. In Singapore, one single peak was detected in May 2002 while two peaks were observed in the other years from 2001 to 2007 [7]. In Hong Kong, two peaks per year were detected between 2001 to 2009 [6]. In mainland China, the seasonality depends on the region. Some provinces [28], [44] have a single peak per year while others have two [27], [44]. Our data fall into the latter group. Similar to our findings, the epidemic period in mainland China was reported from April to July with the highest peak occurring in April or May [26], [44].

The spatio-temporal scan results are sensitive to the parameter choices related to cluster scaling. A maximum spatial cluster size that is too large would detect a cluster that contains some low risk areas and thus much larger than the true cluster, and significant clusters could be missed using a parameter that is too small [37]. In our study, a large cluster (50% scale) occupies half of the study area and contained a considerable proportion of low risk areas. More small clusters with higher elevation in risk were detected using the 10% and 20% scale setting. These small clusters may produce more usable and informative results to epidemiologist for disease prevention initiatives or etiologic investigations [39], [40].

We found three clusters lasting from 1.2 to 2.5 years located in the capital city (Nanning), an industrial city (Liuzhou), a tourism city (Guilin) and their neighbouring areas along a southwestern- northeastern belt. These cities are located on the major transit centers of highways and railways in Guangxi. This finding is consistent with previous reports that clusters are more commonly observed in areas of high population density and mobility [6], [45], [46], which also indicates that the disease may be transmitted along highways and railways. The yearly scan results show that most of the clusters lasted for 2–3 months and occurred in the first of the two peaks during the year, which is consistent with a previous study at the provincial level [26] but the duration were shorter than a study from GuangDong [27]. Although the center of the most likely cluster varied every year, the locations of clusters were concentrated in the southwestern, central and northeastern parts of the province.

The number of severe cases in each year fluctuated based on the predominant pathogen. Varying sizes of clusters were also detected in each year. These findings are different from many previous descriptive studies which reported that severe cases were distributed sporadically [47]. Lack of proper spatio-temporal analysis makes it difficult to reveal clustering for relatively rare events.

The limitation of this study is in the nature of the circular spatial scan statistic, which does not allow for irregular geographic shapes. In addition, as HFMD was in increasing trend, the small clusters in the early years could not be detected when data from all 66 months were used. We remedied this limitation by scanning each year separately, which has the disadvantage of losing information of each year on the continuation of the clusters. The data set used in this study also has some limitations. The sensitivity and positive predictive value of the HFMD surveillance systems in Guangxi and the other provinces in China have not been assessed. The surveillance system in Guangxi and the whole of China has only been in operation since 2008. A longer period of time for trend analysis is probably needed.

In conclusion, both HFMD cases and severe cases have spatio-temporal clusters. The continuous epidemic in Nanning, Liuzhou, Guilin cities and their neighbouring counties as well as moving clusters of severe cases indicate the need to address prevention and control measures in these areas.
